# Clinicopathologic features, genomic profiles and outcomes of younger vs. older Chinese hormone receptor-positive (HR+)/HER2-negative (HER2-) metastatic breast cancer patients

**DOI:** 10.3389/fonc.2023.1152575

**Published:** 2023-06-08

**Authors:** Jinhao Wang, Yaxin Liu, Yuehua Liang, Yue Zhang, Hang Dong, Tiantian Zheng, Jianjun Yu, Pan Du, Shidong Jia, Bonnie L. King, Jing Wang, Xiaoran Liu, Huiping Li

**Affiliations:** ^1^Huidu Shanghai Medical Sciences Ltd., Shanghai, China; ^2^Key Laboratory of Carcinogenesis and Translational Research, Department of Breast Oncology, Peking University Cancer Hospital & Institute, Beijing, China; ^3^Predicine, Inc., Hayward, CA, United States

**Keywords:** hormone receptor-positive breast cancer, metastatic breast cancer, cfDNA, liquid biopsy, biomarkers, prognosis

## Abstract

**Background:**

Poor outcomes have been widely reported for younger vs. older breast cancer patients, but whether this is due to age itself or the enrichment of aggressive clinical features remains controversial. We have evaluated the clinicopathologic characteristics and genomic profiles of real-world hormone receptor-positive (HR+)/HER2-negative (HER2-) metastatic breast cancer (MBC) patients to examine the determinants of outcome for younger vs. older patients in a single clinical subtype undergoing treatment in the same clinic.

**Patients and methods:**

This study included patients presenting at the Peking University Cancer Hospital with primary stage IV or first-line metastatic HR+/HER2- breast cancer who consented to an additional blood draw for genomic profiling prior to treatment. Plasma samples were analyzed with a targeted 152-gene NGS panel to assess somatic circulating tumor DNA (ctDNA) alterations. Genomic DNA (gDNA) extracted from peripheral blood mononuclear cells was analyzed for germline variants using a targeted 600-gene NGS panel. Kaplan-Meier survival analysis was performed to analyze disease free survival (DFS), progression free survival (PFS) and overall survival (OS) in association with clinicopathologic and genomic variables.

**Results:**

Sixty-three patients presenting with HR+/HER2- MBC were enrolled in this study. Fourteen patients were < 40 years, 19 were 40-50 years, and 30 were > 50 years at the time of primary cancer diagnosis. No significant associations were observed between age and DFS, PFS or OS. Shorter OS was associated with *de novo* Stage IV disease (p = 0.002), Luminal B subtype (p = 0.006), high Ki67 index (p = 0.036), resistance to adjuvant endocrine therapy (p = 0.0001) and clinical stage (p = 0.015). Reduced OS was also observed in association with somatic alterations in *FGFR1* (p = 0.008), *CCND2* (p = 0.012), *RB1* (p = 0.029) or *TP53* (p = 0.029) genes, but not in association with germline variants.

**Conclusion:**

In this group of real-world HR+/HER2- MBC breast cancer patients younger age was not associated with poor outcomes. While current guidelines recommend treatment decisions based on tumor biology rather than age, young HR+ breast cancer patients are more likely to receive chemotherapy. Our findings support the development of biomarker-driven treatment strategies for these patients.

## Introduction

Breast cancer is generally a disease of advanced age, with peak incidence occurring after the age of 50 (1, 2). However, while breast cancer is relatively less common before the age of 50, it is the most frequently diagnosed malignancy in younger women worldwide, and a leading cause of cancer deaths in this age group (3). Of the 2.1 million breast cancer cases diagnosed globally in 2018, approximately 645,000 cases and more than 130,000 deaths occurred in women below the age of 50, with incidence and mortality rates varying according to geographic region and socioeconomic levels (4). As a proportion of all breast cancer cases, the highest rates of premenopausal breast cancer incidence and mortality are reported in Africa and Asia (4). Although the greatest burden of premenopausal cases and deaths is observed in countries with a low human development index (HDI), the incidence of premenopausal breast cancer has been climbing in high-income countries (4). Thus, despite being relatively less common, breast cancer in younger women constitutes a significant component of the global breast cancer burden.

Many previous studies have suggested that breast cancer in younger women is a more aggressive disease compared to breast cancer in older women (reviewed) (5–7). Young age at diagnosis has been reported to be an independent risk factor for worse outcomes in many studies, with young patients more likely to die of breast cancer relative to older patients (8–17). Compared to older breast cancer patients, younger breast cancer patients have been identified as more likely to present with *de novo* stage IV disease (11, 16). Young women who present with earlier stage breast cancer have also been reported as more likely to progress to metastatic disease and are more likely to develop brain metastases (18, 19). Studies have also found that young breast cancer patients are more likely to be diagnosed with larger, higher-grade tumors, a higher Ki67 index and positive axillary nodes (8, 10–12, 16, 18, 20, 21). Relative to older breast cancer patients, younger patients have also been characterized for a higher incidence of the more aggressive TNBC and HER2+ clinical subtypes (8, 9, 17, 18, 21–24), and a higher likelihood of harboring *BRCA*1/2 germ line mutations (24–26). In addition, comprehensive genomic profiling, which enables the identification of tumor-related alterations, has demonstrated that younger breast cancer patients have distinct somatic genomic profiles, methylation patterns and gene expression signatures associated with the dysregulation of genes including *ESR1*, *GATA3* and *FGFR1 (* 24, 27–29). Thus, as a group, young breast cancer patients appear to harbor a variety of clinical, biological and genetic features consistent with a more aggressive form of disease.

However, despite the enrichment of TNBC and HER2+ subtypes in younger breast cancer patients, similar to older patients, the majority of cases are HR+/HER2- (9, 17, 18, 21, 23, 24, 27). Moreover, while germline mutations in *BRCA*1/2 are found with higher frequency among younger vs. older patients, the majority of younger patients are not *BRCA*1/2 mutation carriers (25, 30). On the other hand, menopausal status across younger breast cancer patients varies considerably as there is wide deviation around the worldwide average age of menopause at 50 (31). Despite this variation, age is often used as a proxy for menopausal status in epidemiologic and clinical studies (4, 32). Thus, the overlap and variable presence of features across younger breast cancer patients complicates the issue of whether breast cancer in younger patients represents a distinct biologic entity that drives aggressive disease with poor outcomes, or alternatively, reflects a variable enrichment of unfavorable clinical features (17). Two studies focusing solely on HER2+ (33) and TNBC (34) breast cancer patients did not find that young age was associated with poor outcomes. A refined understanding of these issues is needed to optimize management paradigms for young breast cancer patients.

In the present study we have characterized a cohort of 63 real world HR+ Chinese metastatic breast cancer patients undergoing treatment in a single clinic to compare clinical outcomes in association with age and other clinicopathologic features, treatment regimens and genomic profiles to address whether younger patients experience worse outcomes relative to older breast cancer patients.

## Materials and methods

### Patients

**Figure 1:**
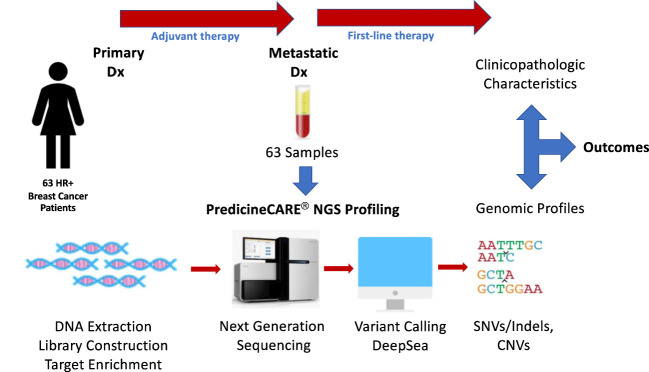
Study design. Plasma samples were collected from 63 HR+/Her2- MBC patients and subjected to NGS profiling using a targeted 152-gene panel to detect somatic mutations and copy number variations. Outcomes were analyzed in association with patient clinicopathological characteristics, treatment regimens and genomic profiles.

Patients presenting at the Peking University Cancer Hospital with first-line metastatic or primary stage IV, HR+/HER2- breast cancer from December 2015 –March 2019 who consented to an additional blood draw for genomic profiling were enrolled in this study. This study was approved by an Institutional Review Board (ethic No. 2016KT75 and ethic No.2017KT40), and all patients signed written informed consent for additional blood collection for genomic profiling. Blood samples (10mL) were prospectively collected from 63 patients, before any treatment was initiated in the metastatic setting (**Figure 1**). Criteria for enrollment included a pathologically confirmed diagnosis of metastatic relapse or *de novo* metastatic disease, with classification of the primary tumor as HR+/HER2-, as confirmed by immunohistochemical staining. HR+ was defined as ≥1% positive staining for tumor nuclear estrogen receptor (ER) and/or progesterone receptor (PR). HER2 staining was evaluated using a range of (0-3+), with 0 and 1+ classified as negative, 2+ as equivocal, and 3+ as positive. Fluorescence *in-situ* hybridization tests were used to confirm HER2 status when immunohistochemistry results were equivocal. Menopausal status at the time of primary breast cancer diagnosis was determined for a subset of patients using the following criteria to define the postmenopausal state: *i)* prior bilateral oophorectomy, *ii)* age > 60 years *iii)* age < 60 years and amenorrheic for 12 or more months in the absence of chemotherapy, tamoxifen, toremifene, or ovarian suppression with follicle-stimulating hormone (FSH) and estradiol levels in the postmenopausal ranges, and *iv)* if taking tamoxifen or toremifene, and age < 60 years, with FSH and plasma estradiol levels in the postmenopausal ranges. Endocrine resistance was defined by a relapse within 2 years of adjuvant endocrine treatment or disease progression during the first 6 months of first-line endocrine therapy for MBC per EMSO 2020 guidelines (35).

### Plasma cfDNA next generation sequencing

Cell-free DNA (cfDNA) testing was performed at the College of American Pathologists (CAP) accredited laboratory at Huidu Shanghai Medical Sciences, Ltd. using the harmonized 152-gene PredicineCARE™ NGS hybrid capture-based sequencing assay. The list of genes covered by this panel is shown in **Supplementary Figure 1**. The details of sample processing and profiling, including library preparation, amplification, hybrid capture and sequencing are described in **Supplementary Methods** and have been described previously (36). In brief, cfDNA was extracted from plasma samples using the QIAamp circulating nucleic acid kit, after which the quantity and quality of purified cfDNA were assessed using a Qubit fluorometer and Bioanalyzer 2100. Five to 30 ng of extracted cfDNA was prepared for library construction in a sequential process involving end-repair, dA-tailing, adapter ligation, and PCR amplification. Amplified DNA libraries with sufficient yields were advanced to hybrid capture with PredicineCARE™ panel probes. Purified libraries underwent quality control analysis with a Bioanalyzer 2100 and were then subjected to paired-end 2x150bp sequencing using the Illumina NGS platform.

### Variant calling

Sequencing data were analyzed using Predicine’s in-house analysis pipeline, which encompasses the initial analysis of raw sequencing data and culminates in final mutation calling, as described in **Supplementary Methods** and a previous publication (36). Briefly, the process included adapter trimming, barcode checking, and correction. Cleaned, paired FASTQ files generated by the pipeline were further aligned to the human reference genome build hg19 using the Burrows-Wheeler Aligner (BWA) (version 0.7.15) alignment tool. Consensus binary alignment map (BAM) files were then derived by merging paired-end reads originated from the same molecules (based on mapping location and unique molecular identifiers) as single strand fragments. Single strand fragments from complementary double strand DNA molecules were further merged as double stranded. Both sequencing and PCR errors were deeply suppressed during this process. Candidate variants, consisting of single nucleotide variants (SNVs), small insertions and deletions (Indels), and copy number variations (CNVs) were identified across the targeted regions covered by the panel. The data analysis was conducted in China.

### Germline mutational status

Genomic DNA (gDNA) was extracted from peripheral blood monocytes and was analyzed for the presence of cancer-associated germline variants with NGS-based sequencing using the comprehensive 600-cancer gene PredicineATLAS™ panel as described in **Supplementary Methods** and a previous publication (37) and to control for mutations resulting from CHIP (Clonal Hematopoiesis of Indeterminant Potential). The data analysis was conducted in China.

### Oncogenic signaling pathway analysis

To examine the proportion of mutations within key oncogenic signaling pathways within the cohort and across age groups, we filtered the list of genes included in a previous publication describing oncogenic signaling pathways (38) to include only those identified as breast cancer driver genes (39, 40), as described in the **Supplementary Methods**. The frequency of SNVs across these genes was analyzed for the entire cohort as well as across age groups, and statistical significance was evaluated using the Fisher’s Exact Test. The data analysis was conducted in China.

### Statistical analysis

Fisher’s exact and Pearson’s chi-squared tests were performed to compare the proportions of patients with defined clinicopathologic variables across age categories and to assess the frequency of genomic alterations across patient subgroups, with significance set at p ≤ 0.05. Kaplan-Meier survival analysis was performed to analyze patient outcomes including disease free survival (DFS), progression free survival (PFS) and overall survival (OS) in association with patient age at the time of primary cancer diagnosis. Outcomes were also analyzed at the univariate level in association with menopausal status, germline status, *de novo* Stage IV disease, type of adjuvant endocrine treatment, treatment with adjuvant chemotherapy, endocrine resistance to adjuvant and relapse therapy, molecular subtype, clinical stage and somatic gene alterations detected at the time of metastatic disease diagnosis. OS was also evaluated in association with age in separate multivariate models that included *de novo* Stage IV disease, molecular subtype, Ki67 index, resistance to adjuvant endocrine therapy and the presence of ctDNA alterations as co-variates using Cox proportional hazards regression. P-values were calculated for univariate and multivariate analyses using the log-rank test, with significance set at p ≤ 0.05. All statistical analyses were performed using R (version 4.1.0).

## Results

### Patient clinicopathologic features across age groups

This study cohort included 63 HR+/HER2- metastatic breast cancer patients who presented at the Peking University Cancer Hospital from December 2015 – March 2019 with metastatic relapse or *de novo* Stage IV metastatic disease. Last follow up was in December 2022. Classification of patients based on clinicopathological characteristics across the three age groups is summarized in **Table 1**.

**Table 1:** Patient clinicopathologic features across age groups.

	All Patients	Patients < 40	Patients 40-50	Patients > 50	p-value
Mean Age at Primary Diagnosis	50.29	33.82	45.96	60.72	
Number of Patients	63	14	19	30	
Menopausal Status at Primary Diagnosis (%)					5.44E-10
Premenopausal	32 (66.67%)*	14 (100%)	17 (94.44%)	1 (6.25%)	
Postmenopausal	16 (33.33%)	0	1 (5.56%)	15 (93.75%)	
Unknown	15				
Histology					0.49
IDC	61 (96.83%)	13 (92.86%)	19(100%)	29 (96.67%)	
ILC	2 (3.17%)	1 (7.14%)	0	1 (3.33%)	
Turnor Grade (%)					0.37
1	6 (9.84%)	1 (7.69%)	2(11.11%)	3 (10%)	
2	40 (65.57%)	6 (46.15%)	12 (66.67%)	22 (73.33%)	
3	15 (24.59%)	6 (46.15%)	4 (22.22%)	5 (16.67%)	
Unknown	2				
Tumor Stage (%)					0.03
1	10 (17.24%)	2 (15.38%)	5 (27.78%)	3 (11.11%)	
2	18 (31.03%)	7 (53.85%)	5 (27.78%)	6 (22.2%)	
3	16 (27.59%)	3 (23.08%)	7 (38.89%)	6 (22.22%)	
4	14 (24.14%)	1 (7.69%)	1 (5.56%)	12 (44.44%)	
Unknown	5				
Molecular Subtype (%)					0.04
Luminal A	16 (26.23%)	3 (23.08%)	9 (47.37%)	4 (13.79%)	
Luminal B	45 (73.77%)	10 (76.92%)	10 (52.63%)	25 (86.21%)	
Unknown	2				
Ki 67 Index (%)					0.68
Low(<20%)	20 (33.33%)	3 (23.08%)	7 (38.89%)	10 (34.48%)	
High P 20%)	40 (66.67%)	10 (76.92%)	11 (61.11%)	19 (65.52%)	
Unknown	3				
De Novo Stage IV (%)	14 (22.22%)	1 (7.14%)	1 (5.26%)	12 (40%)	<0.01
Genaline Alteration Status (%)	16 (25.4%)	5 (35.71%)	4 (21.05%)	7 (23.33%)	0.65
Adjuvant Chemothrapy (%)					0.06
Yes	38 (77.55%)	10 (76.92%)	17 (94.44%)	11 (61.11%)	
No	11 (22.45%)	3 (23.03%)	1 (15.56%)	7 (38.89%)	
Adjuvant Endocrine Therapy (%)					0.04
Yes	43 (87.76%)	12 (92.31%)	18 (100%)	13 (72.22%)	
No	6 (12.24%)	1 (7.69%)	0	5 (27.78%)	
Adjuvant Radiotherapy (%)					0.51
Yes	26 (53.06%)	5 (38.46%)	11 (61.11%)	10 (55.56%)	
No	23 (46.94%)	8 (61.54%)	7 (38.89%)	8 (44.44%)	
DFS (%)					0.84
< 5 yrs	24 (50%)	7 (53.85%)	8 (44.44%)	9 (52.94%)	
>5 yrs	24 (50%)	6 (46.15%)	10 (55.56%)	8 (47.06%)	
Unknown**	1				
Adjuvant Chemotherapy Regimen (%)					0.51
Taxanes	8 (21.05%)	2 (20%)	4 (23.53%)	2 (18.18%)	
Anthracyclines	7 (18.42%)	0	5 (29.41%)	2 (18.18%)	
Both	21 (55.26%)	7 (70%)	7 (41.18%)	7 (63.64%)	
Others	2 (5.26%)	1 (10%)	1 (5.88%)	0	
Endocrine Therapy Only	11				
Adjuvant Endocrine Therapy Regimen (%)					6.87E-05
AI	14 (32.56%)	0	4 (22.22%)	10 (76.92%)	
SERM	29 (67.44%)	12 (100%)	14 (77.78%)	3 (23.08%)	
Chemotherapy Only	6				
Received OFS During Adjuvant Therapy (%)	3 (6.25%)	2 (15.38%)	1 (5.88%)	0	0.35
Chemotherapy Only	6				
First-Line Therapy Regimen (%)					0.73
Chemotherapy Only	12 (19.05%)	3 (21.43%)	5 (26.32%)	4 (13.33%)	
Endocrine Therapy Only	19 (30.16%)	4 (28.57%)	4 (21.05%)	11 (36.67%)	
Both	32 (50.79%)	7(50%)	10 (52.63%)	15 (50%)	
Adjuvant Endocrine Therapy Resistance (%)					0.07
Yes	5 (11.63%)	3(25%)	0	(15.38%)	
No	38 (88.37%)	9 (75%)	18 (100%)	11 (84.62%)	
Did Not Receive Endocrine Therapy	6				
First-Line Endocrine Resistance (%)					0.51
Yes	15 (24.19%)	3 (21.43%)	3 (15.79%)	9 (31.03%)	
No	47 (75.81%)	11 (78.57%)	16 (84.21%)	20 (68.97%)	
Unknown	1				
Number of Metastasis Sites (%)					0.79
≤2 sites	38 (60.32%)	9 (64.29%)	10 (52.63%)	19 (63.33%)	
>2sites	25 (39.68%)	5 (35.71%)	9(47.37%)	1 (36.67%)	
With Visceral Metastasis, no. (%)	33 (52.38%)	6 (42.86%)	12 (63.16%)	15 (50%)	0.48
FGFR1 Mutation, no. (%)	16 (25.40%)	3 (21.43%)	3 (15.79%)	10 (33.33%)	0.39
Median Disease Free Survival (months)	57.05	50.4	71.4	52.7	0.83
Median Progression Free Survival (months)	13.27	12.18	10.23	15.5	0.87
Median Overall Survival (months)	103.97	89.9	127.4	75.5	0.17
Patients Dead Upon Follow-up, no. (%)	40 (63.49%)	9 (64.29%)	11 (57.89%)	20 (66.67%)	0.89

* All percentages based on # of known patients.

**One patient did not undergo surgery due to health issues and was not included in the DFS analysis.

All patients were female and were between the ages of 27 and 82 years (mean = 50.3 years, median = 48.2 years) at the time of primary cancer diagnosis. Fourteen patients were < 40 years of age, 19 patients were between 40-50 years of age, and 30 patients were > 50 years of age. Menopausal status at the time of primary cancer diagnosis was confirmed for 48/63 patients according to the criteria defined in Materials and Methods and varied significantly across age groups (p = 5.44E-10), with the majority of patients under 50 years classified as premenopausal. Further breakdown of menopausal status for the three age groups is presented in **Table 1**.

Germline mutations were detected in 11 genes across 16/63 (25.4%) patients. *BRCA2* mutations were detected in 5 patients and *DNMT3A* alterations were detected in 2 patients. *ALOX12B, ATM, BARD1, BRCA1, CHEK2, IDH1, NOTCH3, PMS and RECQL*, mutations were each detected only once. Three patients were characterized for the presence of two germline mutations: (*ATM* and *BRIP1*), (*RAD50* and *PMS2*) and (*IDH1* and *TP53*) *(* 37). Although the proportion trended highest in patients < 40 years, no significant variation in frequencies of germline mutations were observed across the three age groups (**Table 1**).

Significant variation across age groups was observed for other clinicopathological variables, including *de novo* Stage IV disease (p < 0.01), type of adjuvant endocrine therapy (p = 6.87E-05), and molecular subtype classification (p = 0.04). The highest prevalence of *de novo* Stage IV disease was observed in patients > 50 years (40%). Aromatase inhibitors (AIs) were administered as adjuvant endocrine therapy to 76.92% of women above the age of 50 vs. 22.22% of women aged 40-50 years and 0% of women age < 40 years, respectively. The proportion of patients classified as Luminal B was highest in patients > 50 years (86.21%) and < 40 years (76.92%) and lowest in women aged 40-50 years (52.63%). In keeping with the molecular subtype finding, similar, but nonsignificant trends were observed for lower prevalence of other high-risk features among women aged 40-50 years relative to the other age groups.

### Patient outcomes in association with clinicopathologic features

To assess the contributions of clinicopathologic variables that could potentially influence clinical outcomes for patients in this cohort, univariate analyses were performed to evaluate patient outcomes in association with age and menopausal status at the time of primary disease diagnosis, germline mutation status, *de novo* Stage IV disease, endocrine treatment in the adjuvant and first-line settings, molecular subtype, Ki67 index, clinical stage and resistance to adjuvant and relapse endocrine therapy. Disease free survival (DFS) was measured from the time of surgery following primary diagnosis to the time of diagnosis of first-line metastatic relapse, with a median time to metastasis of 57.05 months (**Table 1**). DFS was measured for 48/49 patients with relapsed metastatic disease but was not determined for one patient that did not undergo surgery due to poor health. Progression free survival (PFS) was measured from the time at which treatment for metastatic disease was initiated to the time of metastatic progression, with a median time to progression of 13.27 months (**Table 1**). PFS was measured for 62/63 patients but was not determined for 1 patient for whom follow up could not be obtained. OS was measured from the time of surgery or treatment for *de novo* disease following primary diagnosis to the time of death, with a median OS of 103.97 months (**Table 1**). OS was determined for all patients except the patient who did not undergo surgery at the time of primary diagnosis.

**Figure 2:**
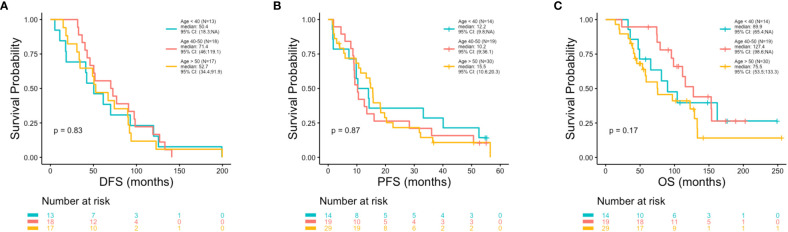
Poor outcomes were not observed in association with younger vs. older age. Kaplan-Meier survival analysis was performed to analyze patient outcomes including disease free survival (DFS), progression free survival (PFS) and overall survival (OS), in association with age. P-values were calculated using the log-rank test, with significance set at p ≤ 0.05. No significant associations were observed between DFS **(A)** or PFS **(B)** or OS **(C)** with age.

**Figure 3:**
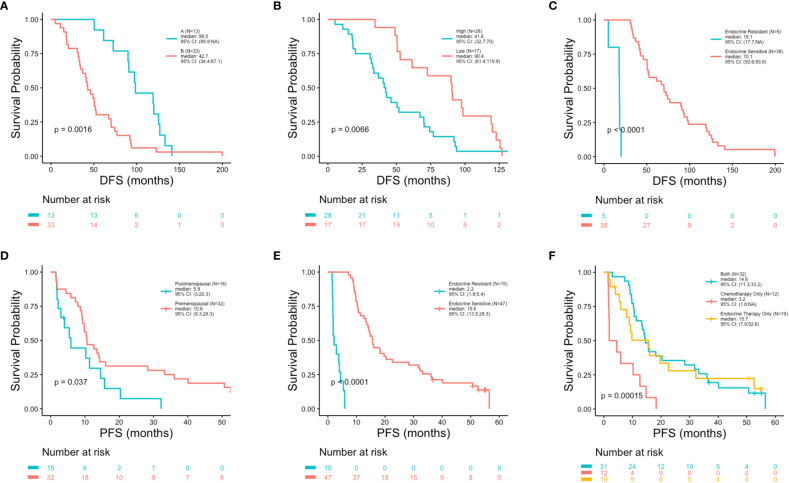
Shorter disease free survival and progression free survival in association with clinicopathologic characteristics. Kaplan-Meier survival analysis was performed to analyze disease free survival (DFS) and progression free survival (PFS) in association with clinicopathologic features. Shorter DFS was observed in associations with luminal subtype (p = 0.0016) **(A)**, high Ki67 index (p = 0.0066) **(B)** and resistance to adjuvant endocrine therapy (p = 0.0001) **(C)**. Shorter PFS was observed in association with postmenopausal status at primary cancer diagnosis (p = 0.037) **(D)**, resistance to relapse endocrine therapy (p = 0.0001) **(E)** and type of first-line therapy regimen (p = 0.00015) **(F)**.

**Figure 4:**
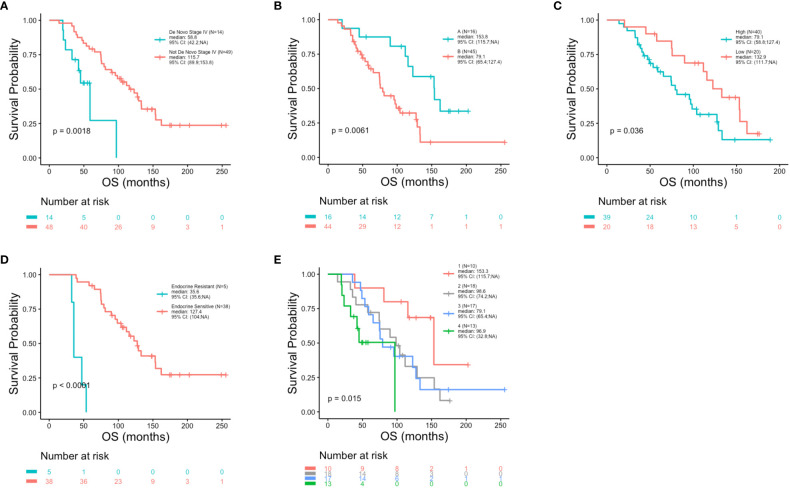
Reduced overall survival in association with clinicopathologic characteristics. Kaplan-Meier survival analysis was performed to analyze overall survival (OS) in association with clinicopathological features. Reduced OS was observed in association with *de novo* stage IV disease (p = 0.0018) **(A)**, luminal B molecular subtype (p = 0.0061) **(B)**, high Ki67 index (p = 0.036) **(C)**, resistance to adjuvant endocrine therapy (p = 0.0001) **(D)** and clinical stage (p = 0.015) **(E)**.

At the univariate level no significant associations were observed between DFS (p = 0.83), PFS (p = 0.87) or OS (p = 0.17) with age (**Figures 2A–C**). Median OS was 89.9, 127.4 and 75.5 months for women <40, 40-50 and >50 years, respectively (**Table 1**). A similar pattern across age groups was observed for DFS (50.4, 71.4 and 52.7 months) but not PFS (12.2, 10.2 and 15.5 months) (**Table 1**). No significant association between age and OS was observed in separate multivariate analyses after adjustment for *de novo* stage IV disease, ctDNA alterations, luminal B subtype, resistance to adjuvant therapy or high Ki67 index (**Table 2**). However, a number of associations were observed between outcomes and several other clinicopathologic variables. Significantly shorter DFS was observed in association with the Luminal B subtype (p = 0.0016) (**Figure 3A**), high Ki67 index (p = 0.007) (**Figure 3B**), and resistance to adjuvant endocrine therapy (p = 0.0001) (**Figure 3C**). Significantly shorter PFS was observed in association with postmenopausal status at the time of primary diagnosis (p = 0.037) (**Figure 3D**), endocrine resistance to relapse endocrine therapy (p = 0.0001) (**Figure 3E**), and type of first-line therapy (p = 0.0002) (**Figure 3F**). No significant associations were observed between postmenopausal status and DFS or OS (**Supplementary Figure 2**). Shorter OS was observed in association with *de novo* Stage IV disease (p = 0.0018) (**Figure 4A**), luminal B molecular subtype (p = 0.0061) (**Figure 4B**), high Ki67 index (p = 0.036) (**Figure 4C**), resistance to adjuvant endocrine therapy (p = 0.0001) (**Figure 4D**) and clinical stage at primary diagnosis (p = 0.015) (**Figure 4E**). No significant associations were observed between any survival outcomes and germline mutation status (**Supplementary Figure 2**), type of adjuvant endocrine therapy or receipt of adjuvant chemotherapy (**Supplementary Figure 3**). PFS was not significantly shorter for patients presenting with *de novo* vs. recurrent Stage IV disease (**Supplementary Figure 4**). DFS, PFS and OS were not significantly shorter for patients when *de novo* Stage IV patients were excluded from the analysis (**Supplementary Figure 5**).

**Table 2:** Multivariate analyses.

Variables	HR (95% CI)	p-value
**Age < 40**	0.83 (0.36-1.93)	0.66
**Age 40-50**	0.64 (0.28-1.44)	0.28
**De Novo Stage IV**	3.11 (1.20-8.07)	0.02
		
		
**Age <40**	0.64 (0.28-1.45)	0.28
**Age 40-50**	0.55 (0.26-1.18)	0.12
**FGFR1 Altered**	2.33 (1.15-4.72)	0.02
		
		
**Age <40**	0.93 (0.41-2.11)	0.86
**Age 40-50**	0.70 (0.30-1.64)	0.41
**Luminal B**	2.48 (1.01-6.10)	0.05
		
		
**Age <40**	0.71 (0.28-1.84)	0.49
**Age 40-50**	0.68 (0.27-1.73)	0.42
**Adjuvant Endocrine Resistance**	35.95 (6.53-197.88)	3.85E-05
		
		
**Age <40**	0.65 (0.29-1.46)	0.29
**Age 40-50**	0.46 (0.21 -1)	0.05
**High Ki67 Status**	2.04 (1.02-4.08)	0.04

Overall survival (OS) was also evaluated in association with age in separate multivariate models that included de novo Stage IV disease, the presence of FGFR1 alterations, molecular subtype, resistance to adjuvant endocrine therapy and high Ki67 index as co-variates using Cox proportional hazards regression. In keeping with univariate analysis, OS was not significantly associated with age.

### ctDNA alterations in association with patient clinicopathologic features

**Figure 5:**
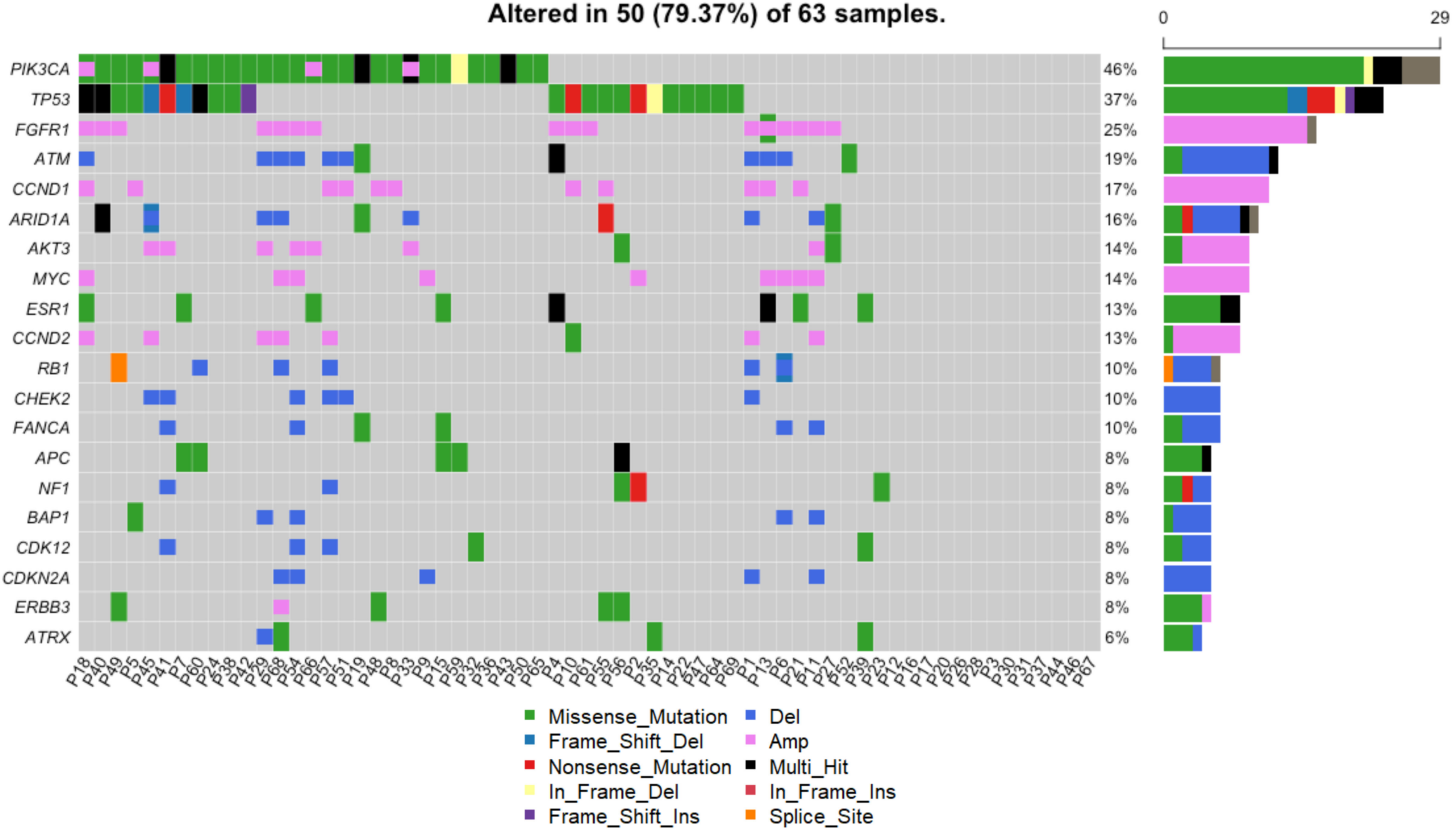
Genomic landscape across all patients. Alterations including SNVs and CNVs were detected in 50/63 (79%) of patients. The heatmap shows alterations of the top 20 most frequently altered genes. Each column shows the alterations for a given patient. The frequency and absolute counts of gene alterations across all patients are shown to the right of the heatmap.

**Figure 6:**
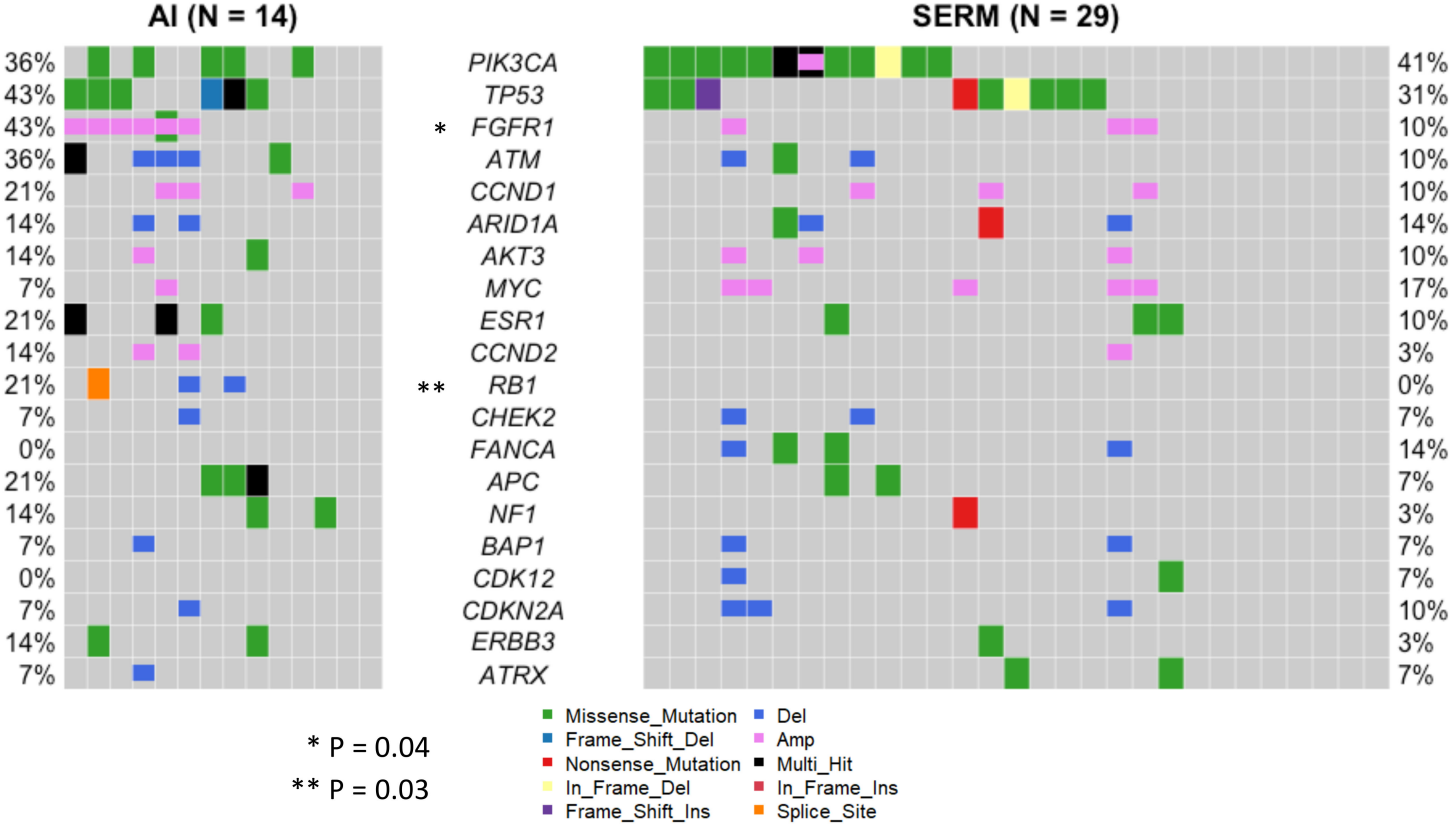
Landscape of genomic alterations across clinicopathological subgroups. Somatic genomic profiles were compared across patients grouped according to age at primary cancer diagnosis, menopausal status at primary cancer diagnosis, germline mutation status, type of adjuvant endocrine therapy, molecular subtype, Ki67 index and tumor grade. Patients who received AI adjuvant endocrine therapy (N=14) had a significantly higher prevalence of *FGFR1* (p = 0.04), and *RB1* (p = 0.03) alterations than patients treated with SERMS (N=29). No significant differences in gene alteration frequencies were observed across age groups, menopausal status, germline mutation status, molecular subtype, Ki67 index, or tumor grade.

Blood samples were collected from 63 patients at the time of metastatic disease diagnosis, prior to the initiation of treatment (**Figure 1**). As such, the mutational profiles detected in these samples reflect the process of primary breast cancer progression and the impact of adjuvant therapies. The landscape of genomic alterations for the entire cohort shows that the top 10 most frequently altered genes across all patients at this timepoint were *PIK3CA* (46%), *TP53* (37%), *FGFR1* (25%), *ATM* (19%) *CCND1* (17%), *ARID1A* (16%), *AKT3* (14%), *MYC* (14%), *ESR1* (13%) and *CCND2* (13%) (**Figure 5**), similar to previously reported findings for an overlapping cohort (36). To examine gene alterations in relation to key patient clinicopathologic variables, we compared somatic genomic profiles across patients grouped according to age at primary cancer diagnosis, menopausal status at primary cancer diagnosis, germline mutation status, type of adjuvant endocrine therapy, molecular subtype, Ki67 index and tumor grade. No significant differences in gene alteration frequencies were observed across age groups, menopausal status, germline mutation status, molecular subtype, Ki67 index, or tumor grade. However, comparison of profiles across women who received adjuvant endocrine therapy revealed a significantly higher prevalence of *FGFR1* (p = 0.04), and *RB1* (p = 0.03) alterations in patients treated with AIs vs. SERMS (**Figure 6**).

### Patient outcomes in association with ctDNA alterations

**Figure 7:**
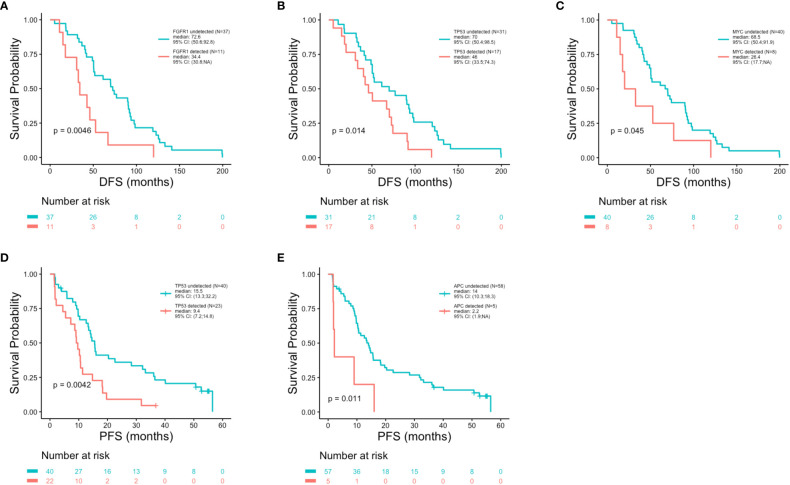
Shorter disease free survival and progression free survival in association with genomic alterations. Kaplan-Meier survival analysis was performed to analyze disease free survival (DFS) and progression free survival (PFS) in association with the top 20 most altered genes. P-values were calculated using the log-rank test, with significance set at p ≤ 0.05. Shorter DFS was associated with the presence of an alteration in *FGFR1* (p = 0.0046) **(A)**, *TP53* (p = 0.014) **(B)** and *MYC* (p = 0.045) **(C)**. Reduced PFS was observed in association with alterations in *TP53* (p = 0.0042) **(D)** and *APC* (p = 0.011) **(E)**.

**Figure 8:**
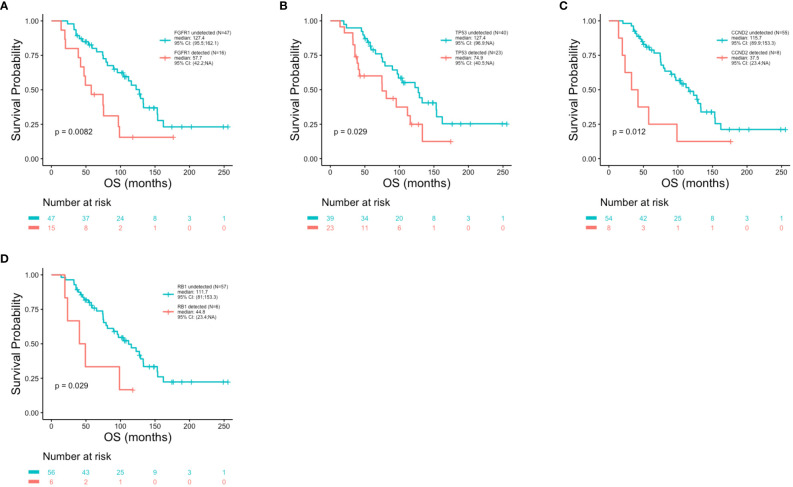
Reduced overall survival in association with genomic alterations. Kaplan-Meier survival analysis was performed to analyze overall survival (OS) in association with specific genomic alterations. P-values were calculated using the log-rank test, with significance set at p ≤ 0.05. Shorter OS was observed in association with *FGFR1* (p = 0.0082) **(A)**, *TP53* (p = 0.029) **(B)**, *CCND2* (p = 0.012) **(C)** and *RB1* (p = 0.029) **(D)** alterations.

Patient outcomes (DFS, PFS and OS) were examined in association with the top 10 most frequent ctDNA alterations (*PIK3CA*, *TP53*, *FGFR1*, *ATM*, *CCND1*, *ARID1A*, *AKT3*, *MYC, ESR1* and *CCND2)* detected across all patients at the time of metastatic disease diagnosis (**Figure 5**). Significantly shorter DFS was observed in association with alterations in *FGFR1* (p = 0.005) (**Figure 7A**), *TP53* (p = 0.014) (**Figure 7B**), and *MYC* (p = 0.045) (**Figure 7C**). Significantly shorter PFS was observed in association with alterations in *TP53* (p = 0.004) (**Figure 7D****)** and *APC* (p = 0.011) (**Figure 7E**). Significantly shorter OS was observed in association with alterations *in FGFR1* (p = 0.008) (**Figure 8A**), *TP53* (p = 0.029) (**Figure 8B**), *CCND2* (p = 0.012) (**Figure 8C**), and *RB1* (p = 0.029) (**Figure 8D**).

### Oncogenic signaling pathway analysis

The proportion of patients in the entire cohort with one or more mutations in a specific oncogenic signaling pathways were: DDR (DNA Damage Response) (60.32%), PI3K (52.38%), TP53 (52.38%), RTK/RAS (23.81%), Cell Cycle (14.29%), HRD (Homologous Recombination Deficiency) (7.94%), Hippo (6.35%) and NOTCH (1.59%). No statistically significant differences were observed across age groups.

## Discussion

Breast cancer in younger women has been widely characterized as a more aggressive form of disease relative to breast cancer in older women (5–7). This has been attributed to a variety of factors including the enrichment of aggressive TNBC and HER2+ clinical subtypes among young breast cancer patients (8, 9, 17, 18, 21–24). However, the majority of young breast cancer cases are HR+ (9, 17, 18, 21, 23, 24, 27). While HR+ breast cancer patients as a group have the most favorable prognosis relative to other clinical subtypes, comparatively poor outcomes have also been reported for younger vs. older HR+ patients (8–10, 13, 14, 17, 41). Breast cancer in younger women is particularly common in China (42), where the average age of diagnosis is 10 years younger than in the U.S (43). In the current study half of the real-world HR+/HER- MBC patients were below the age of 50 at the time of primary cancer diagnosis, providing an opportunity to compare their outcomes with the older patients presenting in the same clinic. We have compared clinical outcomes for these patients in association with clinicopathologic features, treatment regimens and genomic profiles. In this study cohort, younger patients did not experience inferior outcomes compared to older patients.

The relationship between age and breast cancer outcomes is complex and is complicated by a number of factors. First, the cutoff for delineating younger vs. older breast cancer patients varies widely across studies, ranging from <35, < 40, <45 and <50 years (9, 10, 13–17, 44, 45). In the present cohort, we compared outcomes and clinicopathologic variables across three age groups: <40 years, 40-50 years, and >50 years. While no significant differences in survival outcomes were detected across these age groups, we did observe significant enrichment of negative prognostic features and shorter median DFS and OS in women <40 vs. 40-50 years. These trends are consistent with numerous studies reporting worse outcomes for very young (<35-40 years) women vs. women aged 40-50 years (10, 11, 13, 16, 17, 44–46) and suggesting that breast cancer patients below 40 years of age may be clinically distinct from those aged 40-50 years. Relatively poor outcomes for very young women in these studies were attributed to a number of factors including incomplete chemotherapy-induced amenorrhea, nonadherence to treatment, lower ER expression levels, hormone resistance, elevated Ki67 levels and higher tumor grade. Additional variation across studies results from the comparison of data from large population-based registries with results from smaller clinical cohorts, both of which have advantages and disadvantages. While large population-based studies are better powered to detect differences across age groups, they are more likely to encompass lower economic levels that have reduced access to health care, resulting in enrichment of poor prognostic features. Although patients in smaller single center cohorts receive the same care, trends often fail to reach significance. And finally, variable control of key clinicopathologic features has generated variation in results across studies. Several reports suggest that the association of young age with poor outcomes is confined to early-stage breast cancer patients (11, 12, 15, 16), while other studies controlling for or focusing exclusively on specific clinical subtypes have failed to observe an association between young age and poor patient outcomes (22, 33, 34).

In contrast to age, poor outcomes were significantly associated with several other clinicopathologic variables in the present study. Reduced OS was observed in association with *de novo* Stage IV disease, Luminal B subtype, high Ki67 index, resistance to adjuvant endocrine therapy, and clinical stage at primary diagnosis. While a diagnosis of *de novo* Stage IV disease has been reported as more frequent in younger breast cancer patients and has been invoked to support a more aggressive classification of breast cancer diagnosed at young age (11, 16), in the present study *de novo* Stage IV disease was more common in women over the age of 50. As aromatase inhibitors constitute standard of care adjuvant endocrine treatment for postmenopausal women, this therapy regimen was administered to the majority of patients > 50 years, while those in the younger age groups mostly received SERMS (47). While no associations were observed between outcomes and type of adjuvant endocrine therapy, patients treated with AIs had a higher frequency of *FGFR1* alterations, which were significantly associated with poor outcomes.

In this study, genomic profiling was performed at the time of metastatic breast cancer diagnosis, prior to the initiation of treatment for metastatic disease. Thus, the mutational profiles reflect the process of primary breast cancer progression and the impact of adjuvant therapies. Outcomes, including DFS, PFS and OS were examined in association with the top ten most frequent gene alterations observed in the entire study cohort. Poor outcomes were variously associated with *APC*, *CCND2*, *FGFR1, MYC, RB1*, and *TP53* gene alterations. In previous studies *FGFR1* gene alterations have been observed in ~ 7-27% of all breast cancers (48), have been associated with poor prognosis, and constitute a critical mechanism underlying the development of treatment resistance to endocrine suppression *via* AIs and SERMs (48–50). While *FGFR1* alterations, consisting predominantly of gene amplification events, were detected in 25% of all the patients in this study, they were significantly more frequent in women treated with AIs (43%) vs. SERMS (10%). Interestingly, while *FGFR1* alterations were significantly associated with reduced OS and DFS, these alterations were not associated with shorter PFS. These data suggest that the emergence of an *FGFR1* alteration during adjuvant therapy may initiate resistance to adjuvant endocrine therapy that enables accelerated progression to metastatic relapse.

This study has several limitations relating to the small size of the patient cohort, which restricted comparisons and could result in biased analyses. First, due to the small number of patients, it was not possible to evaluate outcomes in association with genomic alterations within age subgroups. Second, due to the small size of the entire cohort, the limited number of events precluded simultaneous evaluation of all pertinent variables within a single multivariate analysis of OS. And third, as adjuvant endocrine therapy was determined by menopausal status, with most patients over 50 receiving AIs, and those under 50 receiving SERMS, it was not possible to incorporate this variable into multivariate analysis. Nevertheless, despite these limitations, all individual multivariate analyses supported the lack of association between OS and age identified in univariate analysis.

In summary, in this study, inferior clinical outcomes were not observed for younger HR+//HER2- metastatic breast cancer patients. Our findings are consistent with other subtype-specific studies that did not find worse outcomes for young HER2+ or TNBC patients (33, 34). While current guidelines advocate that treatment decisions should be based on individual tumor biology rather than age (51), young HR+ breast cancer patients are more likely to receive chemotherapy (52–54). The successful combination of ovarian function suppression with endocrine therapies (55) and the recent inclusion of premenopausal metastatic HR+ breast cancer patients in phase III clinical trials testing CDK4/6 inhibitors clearly demonstrated benefit for young HR+ patients (56), presenting an expanded choice of treatment options. Our findings illustrate the complexity of clinical, biological and genetic variables that underly outcomes for HR+ breast cancer patients of different ages, underscoring the need for biomarker-based treatment strategies for all patients.

## Data availability statement

All data in the main text are publicly available after publication. The supporting data of this study are available on request from the corresponding author (H.L.).

## Ethics statement

This study was approved by an Institutional Review Board (ethic No. 2016KT75 and ethic No.2017KT40), and all patients signed written informed consent for additional blood collection for genomic profiling. The study was performed following Good Clinical Practice and the Declaration of Helsinki under protocols approved by the Ethics Committee of Peking University Cancer Hospital. The patients/participants provided their written informed consent to participate in this study.

## Author contributions

Conception and design of study: BK, HL. Collection of clinical data: YXL, YHL, JGW. Supervision of sample processing, generation and analysis of sequencing data: HL, YZ, HD. Statistical analysis: JHW, TZ. Interpretation of data: JHW, BK, XL, JY, PD, SJ. Drafting of the manuscript: BK. Critical revision of the manuscript for important intellectual data: BK, SJ, HL. Obtaining funding: HL. All authors contributed to the article and approved the submitted version.

## References

[1] DeSantis CE Ma J Gaudet MM Newman LA Miller KD Goding Sauer A. Breast cancer statistics, 2019. CA Cancer J Clin (2019) 69(6):438–51. doi: 10.3322/caac.21583 31577379

[2] Lei S Zheng R Zhang S Wang S Chen R Sun K. Global patterns of breast cancer incidence and mortality: a population-based cancer registry data analysis from 2000 to 2020. Cancer Commun (Lond) (2021) 41(11):1183–94. doi: 10.1002/cac2.12207 PMC8626596 34399040

[3] Fidler MM Gupta S Soerjomataram I Ferlay J Steliarova-Foucher E Bray F. Cancer incidence and mortality among young adults aged 20-39 years worldwide in 2012: a population-based study. Lancet Oncol (2017) 18(12):1579–89. doi: 10.1016/S1470-2045(17)30677-0 29111259

[4] Heer E Harper A Escandor N Sung H McCormack V Fidler-Benaoudia MM. Global burden and trends in premenopausal and postmenopausal breast cancer: a population-based study. Lancet Glob Health (2020) 8(8):e1027–e37. doi: 10.1016/S2214-109X(20)30215-1 32710860

[5] Azim HA Jr. Partridge AH. Biology of breast cancer in young women. Breast Cancer Res (2014) 16(4):427. doi: 10.1186/s13058-014-0427-5 25436920 PMC4303229

[6] Assi HA Khoury KE Dbouk H Khalil LE Mouhieddine TH El Saghir NS. Epidemiology and prognosis of breast cancer in young women. J Thorac Dis (2013) 5 Suppl 1:S2–8. doi: 10.3978/j.issn.2072-1439.2013.05.24 PMC3695538 23819024

[7] Johnson RH Anders CK Litton JK Ruddy KJ Bleyer A. Breast cancer in adolescents and young adults. Pediatr Blood Cancer. (2018) 65(12):e27397. doi: 10.1002/pbc.27397 30156052 PMC6192832

[8] Azim HA Jr. Michiels S Bedard PL Singhal SK Criscitiello C Ignatiadis M. Elucidating prognosis and biology of breast cancer arising in young women using gene expression profiling. Clin Cancer Res (2012) 18(5):1341–51. doi: 10.1158/1078-0432.CCR-11-2599 22261811

[9] Cancello G Maisonneuve P Rotmensz N Viale G Mastropasqua MG Pruneri G. Prognosis and adjuvant treatment effects in selected breast cancer subtypes of very young women (<35 years) with operable breast cancer. Ann Oncol (2010) 21(10):1974–81. doi: 10.1093/annonc/mdq072 20332136

[10] El Saghir NS Seoud M Khalil MK Charafeddine M Salem ZK Geara FB. Effects of young age at presentation on survival in breast cancer. BMC Cancer (2006) 6:194. doi: 10.1186/1471-2407-6-194 16857060 PMC1555600

[11] Fredholm H Magnusson K Lindstrom LS Garmo H Falt SE Lindman H. Long-term outcome in young women with breast cancer: a population-based study. Breast Cancer Res Treat (2016) 160(1):131–43. doi: 10.1007/s10549-016-3983-9 PMC5050247 27624330

[12] Gnerlich JL Deshpande AD Jeffe DB Sweet A White N Margenthaler JA. Elevated breast cancer mortality in women younger than age 40 years compared with older women is attributed to poorer survival in early-stage disease. J Am Coll Surg (2009) 208(3):341–7. doi: 10.1016/j.jamcollsurg.2008.12.001 PMC3262236 19317994

[13] Han W Kang SY Korean Breast Cancer S. Relationship between age at diagnosis and outcome of premenopausal breast cancer: age less than 35 years is a reasonable cut-off for defining young age-onset breast cancer. Breast Cancer Res Treat (2010) 119(1):193–200. doi: 10.1007/s10549-009-0388-z 19350387

[14] Arvold ND Taghian AG Niemierko A Abi Raad RF Sreedhara M Nguyen PL. Age, breast cancer subtype approximation, and local recurrence after breast-conserving therapy. J Clin Oncol (2011) 29(29):3885–91. doi: 10.1200/JCO.2011.36.1105 PMC3189090 21900114

[15] Kroman N Jensen MB Wohlfahrt J Mouridsen HT Andersen PK Melbye M. Factors influencing the effect of age on prognosis in breast cancer: population based study. BMJ (2000) 320(7233):474–8. doi: 10.1136/bmj.320.7233.474 PMC27289 10678859

[16] Kataoka A Iwamoto T Tokunaga E Tomotaki A Kumamaru H Miyata H. Young adult breast cancer patients have a poor prognosis independent of prognostic clinicopathological factors: a study from the Japanese breast cancer registry. Breast Cancer Res Treat (2016) 160(1):163–72. doi: 10.1007/s10549-016-3984-8 PMC5050233 27647460

[17] Sheridan W Scott T Caroline S Yvonne Z Vanessa B David V. Breast cancer in young women: have the prognostic implications of breast cancer subtypes changed over time? Breast Cancer Res Treat (2014) 147(3):617–29. doi: 10.1007/s10549-014-3125-1 25209005

[18] Akrami M Sepahdar A Arasteh P Tahmasebi S Zangouri V Askari A. Do site and type of metastasis in breast cancer show a changing pattern with increased age? a cross comparison of clinicopathological characteristics between age groups. World J Surg Oncol (2018) 16(1):147. doi: 10.1186/s12957-018-1435-1 30025533 PMC6053760

[19] Hung MH Liu CY Shiau CY Hsu CY Tsai YF Wang YL. Effect of age and biological subtype on the risk and timing of brain metastasis in breast cancer patients. PloS One (2014) 9(2):e89389. doi: 10.1371/journal.pone.0089389 24586742 PMC3933537

[20] Anders CK Hsu DS Broadwater G Acharya CR Foekens JA Zhang Y. Young age at diagnosis correlates with worse prognosis and defines a subset of breast cancers with shared patterns of gene expression. J Clin Oncol (2008) 26(20):3324–30. doi: 10.1200/JCO.2007.14.2471 18612148

[21] Plichta JK Thomas SM Vernon R Fayanju OM Rosenberger LH Hyslop T. Breast cancer tumor histopathology, stage at presentation, and treatment in the extremes of age. Breast Cancer Res Treat (2020) 180(1):227–35. doi: 10.1007/s10549-020-05542-4 PMC7066434 31980967

[22] Anders CK Fan C Parker JS Carey LA Blackwell KL Klauber-DeMore N. Breast carcinomas arising at a young age: unique biology or a surrogate for aggressive intrinsic subtypes? J Clin Oncol (2011) 29(1):e18–20. doi: 10.1200/JCO.2010.28.9199 PMC3055864 21115855

[23] Loibl S Jackisch C Lederer B Untch M Paepke S Kummel S. Outcome after neoadjuvant chemotherapy in young breast cancer patients: a pooled analysis of individual patient data from eight prospectively randomized controlled trials. Breast Cancer Res Treat (2015) 152(2):377–87. doi: 10.1007/s10549-015-3479-z 26109347

[24] Kan Z Ding Y Kim J Jung HH Chung W Lal S. Multi-omics profiling of younger Asian breast cancers reveals distinctive molecular signatures. Nat Commun (2018) 9(1):1725. doi: 10.1038/s41467-018-04129-4 29713003 PMC5928087

[25] Ponder BAJ Day NE Easton DF Pharoah PDP Lipscombe JM Redman K Prevalence and penetrance of BRCA1 and BRCA2 mutations in a population-based series of breast cancer cases. anglian breast cancer study group. Br J Cancer (2000) 83(10):1301–8. doi: 10.1054/bjoc.2000.1407 PMC2408797 11044354

[26] Malone KE Daling JR Doody DR Hsu L Bernstein L Coates RJ. Prevalence and predictors of BRCA1 and BRCA2 mutations in a population-based study of breast cancer in white and black American women ages 35 to 64 years. Cancer Res (2006) 66(16):8297–308. doi: 10.1158/0008-5472.CAN-06-0503 16912212

[27] Azim HA Jr. Nguyen B Brohee S Zoppoli G Sotiriou C. Genomic aberrations in young and elderly breast cancer patients. BMC Med (2015) 13:266. doi: 10.1186/s12916-015-0504-3 26467651 PMC4606505

[28] Liao S Hartmaier RJ McGuire KP Puhalla SL Luthra S Chandran UR. The molecular landscape of premenopausal breast cancer. Breast Cancer Res (2015) 17:104. doi: 10.1186/s13058-015-0618-8 26251034 PMC4531812

[29] Siddig A Tengku Din T Mohd Nafi SN Yahya MM Sulong S Wan Abdul Rahman WF. The unique biology behind the early onset of breast cancer. Genes (Basel). (2021) 12(3):1-21. doi: 10.3390/genes12030372 PMC8000244 33807872

[30] Copson ER Maishman TC Tapper WJ Cutress RI Greville-Heygate S Altman DG. Germline BRCA mutation and outcome in young-onset breast cancer (POSH): a prospective cohort study. Lancet Oncol (2018) 19(2):169–80. doi: 10.1016/S1470-2045(17)30891-4 PMC5805863 29337092

[31] Schoenaker DA Jackson CA Rowlands JV Mishra GD. Socioeconomic position, lifestyle factors and age at natural menopause: a systematic review and meta-analyses of studies across six continents. Int J Epidemiol. (2014) 43(5):1542–62. doi: 10.1093/ije/dyu094 PMC4190515 24771324

[32] Shah AN Carroll KJ Gerratana L Lin C Davis AA Zhang Q. Circulating tumor cells, circulating tumor DNA, and disease characteristics in young women with metastatic breast cancer. Breast Cancer Res Treat (2021) 187(2):397–405. doi: 10.1007/s10549-021-06236-1 34076801

[33] Partridge AH Gelber S Piccart-Gebhart MJ Focant F Scullion M Holmes E. Effect of age on breast cancer outcomes in women with human epidermal growth factor receptor 2-positive breast cancer: results from a herceptin adjuvant trial. J Clin Oncol (2013) 31(21):2692–8. doi: 10.1200/JCO.2012.44.1956 23752109

[34] Aine M Boyaci C Hartman J Hakkinen J Mitra S Campos AB. Molecular analyses of triple-negative breast cancer in the young and elderly. Breast Cancer Res (2021) 23(1):20. doi: 10.1054/bjoc.2000.1407 33568222 PMC7874480

[35] Cardoso F Paluch-Shimon S Senkus E Curigliano G Aapro MS Andre F. 5th ESO-ESMO international consensus guidelines for advanced breast cancer (ABC 5). Ann Oncol (2020) 31(12):1623–49. doi: 10.1016/j.annonc.2020.09.010 PMC7510449 32979513

[36] Liu X Davis AA Xie F Gui X Chen Y Zhang Q. Cell-free DNA comparative analysis of the genomic landscape of first-line hormone receptor-positive metastatic breast cancer from the US and China. Breast Cancer Res Treat (2021) 190(2):213–26. doi: 10.1007/s10549-021-06370-w PMC8558197 34471951

[37] Zhang J Wang N Zheng T Lu T Zhang R Ran R. Germline mutational landscape in Chinese patients with advanced breast cancer. Front Oncol (2022) 12:745796. doi: 10.3389/fonc.2022.745796 35494038 PMC9043949

[38] Sanchez-Vega F Mina M Armenia J Chatila WK Luna A La KC. Oncogenic signaling pathways in the cancer genome atlas. Cell (2018) 173(2):321–37 e10. doi: 10.1016/j.cell.2018.03.035 29625050 PMC6070353

[39] Dietlein F Weghorn D Taylor-Weiner A Richters A Reardon B Liu D. Identification of cancer driver genes based on nucleotide context. Nat Genet (2020) 52(2):208–18. doi: 10.1038/s41588-019-0572-y PMC7031046 32015527

[40] Martinez-Jimenez F Muinos F Sentis I Deu-Pons J Reyes-Salazar I Arnedo-Pac C. A compendium of mutational cancer driver genes. Nat Rev Cancer. (2020) 20(10):555–72. doi: 10.1038/s41568-020-0290-x 32778778

[41] Sun H Huang W Ji F Pan Y Yang L. Comparisons of metastatic patterns, survival outcomes and tumor immune microenvironment between young and non-young breast cancer patients. Front Cell Dev Biol (2022) 10:923371. doi: 10.3389/fcell.2022.923371 35912097 PMC9329535

[42] Fan L Strasser-Weippl K Li JJ St Louis J Finkelstein DM Yu KD. Breast cancer in China. Lancet Oncol (2014) 15(7):e279–89. doi: 10.1016/S1470-2045(13)70567-9 24872111

[43] Song QK Li J Huang R Fan JH Zheng RS Zhang BN. Age of diagnosis of breast cancer in china: almost 10 years earlier than in the united states and the European union. Asian Pac J Cancer Prev (2014) 15(22):10021–5. doi: 10.7314/APJCP.2014.15.22.10021 25520063

[44] Ahn SH Son BH Kim SW Kim SI Jeong J Ko SS. Poor outcome of hormone receptor-positive breast cancer at very young age is due to tamoxifen resistance: nationwide survival data in Korea–a report from the Korean breast cancer society. J Clin Oncol (2007) 25(17):2360–8. doi: 10.1200/JCO.2006.10.3754 17515570

[45] Colleoni M Rotmensz N Peruzzotti G Maisonneuve P Orlando L Ghisini R. Role of endocrine responsiveness and adjuvant therapy in very young women (below 35 years) with operable breast cancer and node negative disease. Ann Oncol (2006) 17(10):1497–503. doi: 10.1093/annonc/mdl145 16798834

[46] Chia KS Du WB Sankaranarayanan R Sankila R Wang H Lee J. Do younger female breast cancer patients have a poorer prognosis? results from a population-based survival analysis. Int J Cancer (2004) 108(5):761–5. doi: 10.1002/ijc.11632 14696104

[47] Xu B Li H Jiang Z Gu L Tang J Xie H. Adjuvant tamoxifen switched to exemestane treatment in postmenopausal women with estrogen receptor-positive early breast cancer: a pragmatic, multicenter, and prospective clinical trial in China. Chin J Cancer Res (2022) 34(6):592–600. doi: 10.21147/j.issn.1000-9604.2022.06.07 36714346 PMC9829502

[48] Drago JZ Formisano L Juric D Niemierko A Servetto A Wander SA. FGFR1 amplification mediates endocrine resistance but retains TORC sensitivity in metastatic hormone receptor-positive (HR(+)) breast cancer. Clin Cancer Res (2019) 25(21):6443–51. doi: 10.1158/1078-0432.CCR-19-0138 PMC6825550 31371343

[49] Giltnane JM Hutchinson KE Stricker TP Formisano L Young CD Estrada MV. Genomic profiling of ER(+) breast cancers after short-term estrogen suppression reveals alterations associated with endocrine resistance. Sci Transl Med (2017) 9(402):1-35. doi: 10.1126/scitranslmed.aai7993 PMC5723145 28794284

[50] Turner N Pearson A Sharpe R Lambros M Geyer F Lopez-Garcia MA. FGFR1 amplification drives endocrine therapy resistance and is a therapeutic target in breast cancer. Cancer Res (2010) 70(5):2085–94. doi: 10.1158/0008-5472.CAN-09-3746 PMC2832818 20179196

[51] Paluch-Shimon S Cardoso F Partridge AH Abulkhair O Azim HA Jr. Bianchi-Micheli G. ESO-ESMO 4th international consensus guidelines for breast cancer in young women (BCY4). Ann Oncol (2020) 31(6):674–96. doi: 10.1016/j.annonc.2020.03.284 32199930

[52] Dalal AA Gauthier G Gagnon-Sanschagrin P Burne R Guerin A Niravath P. Treatment and monitoring patterns among premenopausal women with HR+/HER2- advanced breast cancer. Adv Ther (2018) 35(9):1356–67. doi: 10.1007/s12325-018-0764-3 30105655

[53] Hartkopf AD Huober J Volz B Nabieva N Taran FA Schwitulla J. Treatment landscape of advanced breast cancer patients with hormone receptor positive HER2 negative tumors - data from the German PRAEGNANT breast cancer registry. Breast (2018) 37:42–51. doi: 10.1016/j.breast.2017.10.002 29100043

[54] Lobbezoo DJ van Kampen RJ Voogd AC Dercksen MW van den Berkmortel F Smilde TJ. In real life, one-quarter of patients with hormone receptor-positive metastatic breast cancer receive chemotherapy as initial palliative therapy: a study of the southeast Netherlands breast cancer consortium. Ann Oncol (2016) 27(2):256–62. doi: 10.1093/annonc/mdv544 26578730

[55] Francis PA Pagani O Fleming GF Walley BA Colleoni M Lang I. Tailoring adjuvant endocrine therapy for premenopausal breast cancer. N Engl J Med (2018) 379(2):122–37. doi: 10.1056/NEJMoa1803164 PMC6193457 29863451

[56] Shah AN Metzger O Bartlett CH Liu Y Huang X Cristofanilli M. Hormone receptor-Positive/Human epidermal growth receptor 2-negative metastatic breast cancer in young women: emerging data in the era of molecularly targeted agents. Oncologist (2020) 25(6):e900–e8. doi: 10.1634/theoncologist.2019-0729 PMC7288640 32176406

