# Supplementary material for: Clinicopathologic features, genomic profiles and outcomes of younger vs. older Chinese hormone receptor-positive (HR+)/HER2-negative (HER2-) metastatic breast cancer patients
Source: Front Oncol. 2023 Jun 8;13:1152575. doi: 10.3389/fonc.2023.1152575 (PMC10286822; doi:10.3389/fonc.2023.1152575)
Supplement: Supplementary file 1 [file DataSheet_1.pdf]

Supplementary Material

TABLE OF CONTENTS Page

Supplementary Methods 2

References 4

Supplementary Figures 5

Figure S1. PredicineCARE™ gene panel. 6

Figure S2: Outcomes in association with menopausal and germline mutation status. 7

Figure S3. Outcomes in association with type of adjuvant endocrine therapy (AIs vs. SERMS), and receipt of adjuvant chemotherapy. 8

Figure S4. PFS in association with Stage IV vs. de novo Stage IV disease. 9

Figure S5. Outcomes in association with age exclusive of de novo Stage IV patients. 10

1 Supplementary Methods

Patients and study design.

Sixty-three HR+/HER2- metastatic breast cancer patients presenting at the time of metastatic relapse or with *de-novo* Stage IV metastatic disease at the Peking University Cancer Hospital from December 2015 – March 1919 who consented to an additional blood draw for genomic profiling participated in this study. Patient ages ranged from 27-82 years. Blood samples were prospectively collected from all patients, before any treatment was initiated in the metastatic setting. Patients were treated with standard of care first line endocrine therapy including OFS for premenopausal patients. A subset of patients also received first line chemotherapy. Clinical data was collected from December 2015 through the censor date of March 30, 2022. Last follow up was in March 2022. This study was approved by an Institutional Review Board (ethic No. 2016KT75 and ethic No.2017KT40), and all patients signed written informed consent for additional blood collection for cfDNA profiling. The study was performed following Good Clinical Practice and the Declaration of Helsinki under protocols approved by the Ethics Committee of Peking University Cancer Hospital.

Blood collection and cfDNA/gDNA extraction

Each single draw of 10 mL of whole blood was collected into a Streck tube before undergoing a two-step centrifugation to separate plasma and buffy coat compartments. Aliquoted samples were stored at -80°C for batch processing. DNA extraction, library preparation and sequencing were performed in a CAP-accredited laboratory (Huidu Shanghai). Circulating cell-free DNA (cfDNA) was extracted from plasma samples using the QIAamp circulating nucleic acid kit (Qiagen, Hilden, Germany). Quantity and quality of the purified cfDNA were checked using a Qubit fluorimeter (ThermoFisher Scientific, Waltham, Massachusetts, USA) and Bioanalyzer 2100 (Agilent Technologies, California, USA). For cfDNA samples with severe genomic contamination from peripheral blood cells, a bead-based size selection was performed to remove large genomic fragments (AMPure XP beads, Beckman Coulter, California, USA). Genomic DNA (gDNA) was extracted from matched peripheral blood mononuclear cells (PBMCs) using the QIAamp DNA Blood Mini Kit (Qiagen), then enzymatically fragmented and purified.

Library preparation, hybrid capture and sequencing

Five to 30 ng of extracted cfDNA or 30-50 ng of fragmented PBMC gDNA were then processed for library construction including end-repair dA-tailing and adapter ligation. Ligated library fragments with appropriate adapters were amplified via PCR. The amplified DNA libraries were then further checked using a Bioanalyzer 2100 and samples with sufficient yield were advanced to hybrid capture. Hybrid capture was conducted using Biotin labelled DNA probes. In brief, each library was hybridized overnight with a Predicine NGS panel and paramagnetic beads. The unbound fragments were washed away, and the enriched fragments were amplified via PCR amplification. The purified product was checked on a Bioanalyzer 2100 and then loaded into an Illumina NovaSeq 6000 (San Diego, CA, USA) for NGS sequencing with paired-end 2x150bp sequencing kits.

Analyses of NGS data from cfDNA

NGS data from cfDNA were analyzed using the Predicine DeepSea NGS analysis pipeline, which starts from the raw sequencing data (BCL files) and outputs the final mutation calls. Briefly, the pipeline first performed adapter trimming, barcode checking, and correction. Cleaned paired FASTQ files were aligned to human reference genome build hg19 using the BWA alignment tool. Consensus BAM files were then derived by merging paired-end reads originated from the same molecules (based on mapping location and unique molecular identifiers) as single strand fragments. Single strand fragments from the same double strand DNA molecules were further merged as double stranded. By using the error suppression method described in [Newman, 2016], both sequencing and PCR errors were mostly corrected during this process.

Somatic mutation identification

Candidate variants were called by comparing with local variant background (defined based on plasma samples from healthy donors and historical data). Variants were further filtered by log-odds (LOD) threshold [Cibulskis 2013], base and mapping quality thresholds, repeat regions and other quality metrics. Candidate somatic mutations were further filtered on the basis of gene annotation to identify those occurring in protein-coding regions. Intronic and silent changes were excluded, while mutations resulting in missense mutations, nonsense mutations, frameshifts, or splice site alterations were retained. Mutations annotated as benign or likely benign were also filtered out based on the ClinVar database [Landrum, 2016], or as common germline variants in databases including 1000 genomes [Auton, 205; Sudmant, 2015], ExAC [Lek, 2016], gnomAD (http://gnomad.broadinstitute.org) and KAVIAR [Glusman, 20111] with population allele frequency >0.5%. Finally, hematopoietic expansion-related variants that have been previously described, including those in DNMT3A, ASXL1, TET2, and specific alterations within ATM (residue 3008), GNAS (residue 201, 202), or JAK2 (residue 617) were marked as CHIP-related mutations.

Oncogenic Signaling Pathway Analysis

To examine the relative proportion of mutations within key oncogenic signaling pathways in this patient cohort, we filtered the list of genes included in a previous publication describing oncogenic signaling pathways [Sanchez-Vega, 2018] to include only those identified as breast cancer driver genes [Dietlein, 2020; Martinez-Jiminez, 2020]. The list of resulting genes is shown in the table below. The frequency of SNVs across these genes was compared across age groups and statistical significance was evaluated using the Fisher's Exact Test.

Pathway	Gene List
HRD	BRCA1,BRCA2
DDR	BRCA1,TP53,BAP1,BRCA2,PTEN,ATM,ARID1A,POLD1
PI3K	PIK3CA,AKT1,PTEN,PIK3R1,MTOR
Cell_Cycle	RB1,CDKN2A,CDKN1B
TP53	TP53,ATM
RTK-RAS	EGFR,FGFR2,BRAF,NF1,KRAS,ERBB3,ERBB4,JAK2,ALK,PDGFRB,HRAS,ERBB2
Hippo	FAT1,FAT4,FAT3
NOTCH	NOTCH2,CREBBP,NCOR2,FBXW7,SPEN
WNT	
MYC	
NRF2	
TGF-Beta	SMAD2,SMAD4

Germline DNA analysis

Germline variants were determined by concurrent sequencing of buffy coat PBMCs using the PredicineATLAS™ targeted 600-gene panel. Candidate variants with low base quality, mapping scores, and other poor-quality metrics were filtered. Candidate variants with an allelic frequency <5% or with less than 8 distinct reads containing the mutation were excluded. Unknown variants in repeat regions were also excluded. Details of the analytical workflow are provided above in "Analyses of NGS data generated from cfDNA".

References

1) Newman AM, Lovejoy AF, Klass DM *et al.* Integrated digital error suppression for improved detection of circulating tumor DNA. *Nat Biotechnol* 2016; **34**:547-555.
2) Cibulskis K, Lawrence MS, Carter SL *et al.* Sensitive detection of somatic point mutations in impure and heterogeneous cancer samples. *Nat Biotechnol* 2013; **31**:213-219.
3) Landrum MJ, Lee JM, Benson M *et al.* ClinVar: public archive of interpretations of clinically relevant variants. *Nucleic Acids Res* 2016; **44**:D862-868.
4) Auton A, Abecasis GR, Altshuler DM *et al.* A global reference for human genetic variation. *Nature* 2015; **526**:68-74.
5) Sudmant PH, Rausch T, Gardner EJ *et al.* An integrated map of structural variation in 2,504 human genomes. *Nature* 2015; **526**:75-81.
6) Lek M, Karczewski KJ, Minikel EV *et al.* Analysis of protein-coding genetic variation in 60,706 humans. *Nature* 2016; **536**:285-291.
7) Glusman G, Caballero J, Mauldin DE, Hood L, Roach JC. Kaviar: an accessible system for testing SNV novelty. *Bioinformatics* 2011; **27**:3216-3217.

8) Sanchez-Vega F, Mina M, Armenia J, Chatila WK, Luna A, La KC, et al. Oncogenic Signaling Pathways in The Cancer Genome Atlas. Cell. 2018;173(2):321-37 e10.
9) Dietlein F, Weghorn D, Taylor-Weiner A, Richters A, Reardon B, Liu D, et al. Identification of cancer driver genes based on nucleotide context. Nat Genet. 2020;52(2):208-18.
10) Martinez-Jimenez F, Muinos F, Sentis I, Deu-Pons J, Reyes-Salazar I, Arnedo-Pac C, et al. A compendium of mutational cancer driver genes. Nat Rev Cancer. 2020;20(10):555-72.

2 Supplementary Figures

ABRAXAS1	AKT1	AKT2	AKT3	ALK*	APC	AR	ARAF	ARID1A	ATM
ATRX	BAP1	BARD1	BCL2	BRAF	BRCA1	BRCA2	BRIP1	BTK*	CCND1
CCND2*	CCND3	CCNE1*	CCNE2	CD274 (PD-L1)	CD74	CDH1	CDK12	CDK2	CDK4
CDK6	CDKN2A	CHEK1	CHEK2	CTNNB1*	CXCR4	CYP2C19*	CYP2D6*	CYP3A4*	DAXX
DDR2*	DPYD*	E2F1	EGFR	EPCAM*	ERBB2 (HER2)	ERBB3*	ERCC1	ESR1	EZH2
FANCA	FANCC	FANCF	FANCG	FANCL	FAT1*	FBXW7*	FEN1	FGFR1	FGFR2
FGFR3	FGFR4	FLT3*	FOXA1	FOXL2	FZR1	GEN1	GNA11	GNAQ	GNAS*
GSTP1*	HNF1A*	HOXB13	HRAS	IDH1*	IDH2*	JAK2*	JAK3*	KDM6A*	KIT
KMT2C*	KMT2D* (MLL2)	KRAS	MAP2K1 (MEK1)	MAP2K2 (MEK2)	MAPK1	MAPK3	MDM2	MET	MLH1
MPL*	MRE11	MSH2	MSH6	MTHFR*	MTOR*	MYC	MYCN	MYD88	NBN
NF1*	NFE2L2*	NOTCH1*	NPM1*	NRAS	NTRK1	NTRK2	NTRK3	PALB2	PDCD1LG2 (PD-L2)
PDGFRA*	PIK3CA	PIK3CB	PIK3R1	PLCG2*	PMS2	POLD1	POLE	PPP2R1A*	PRKACA*
PRKD1*	PTEN	PTPN11	RAD50	RAD51	RAD51B	RAD51C	RAD51D	RAD52	RAF1
RB1	RET*	RHEB*	RHOA*	RIT1*	RNF43*	ROS1*	SDHB	SMAD4	SMO
SPOP	STAG2	STK11	TERT* promoter	TMPRSS2	TP53	TSC1*	TSC2*	UGT1A1*	VHL
XPC*	XRCC1*								

SNVs + Indels   CNVs   Fusions   Fusions + CNVs

Supplementary Figure 1. PredicineCARE^{TM} gene panel

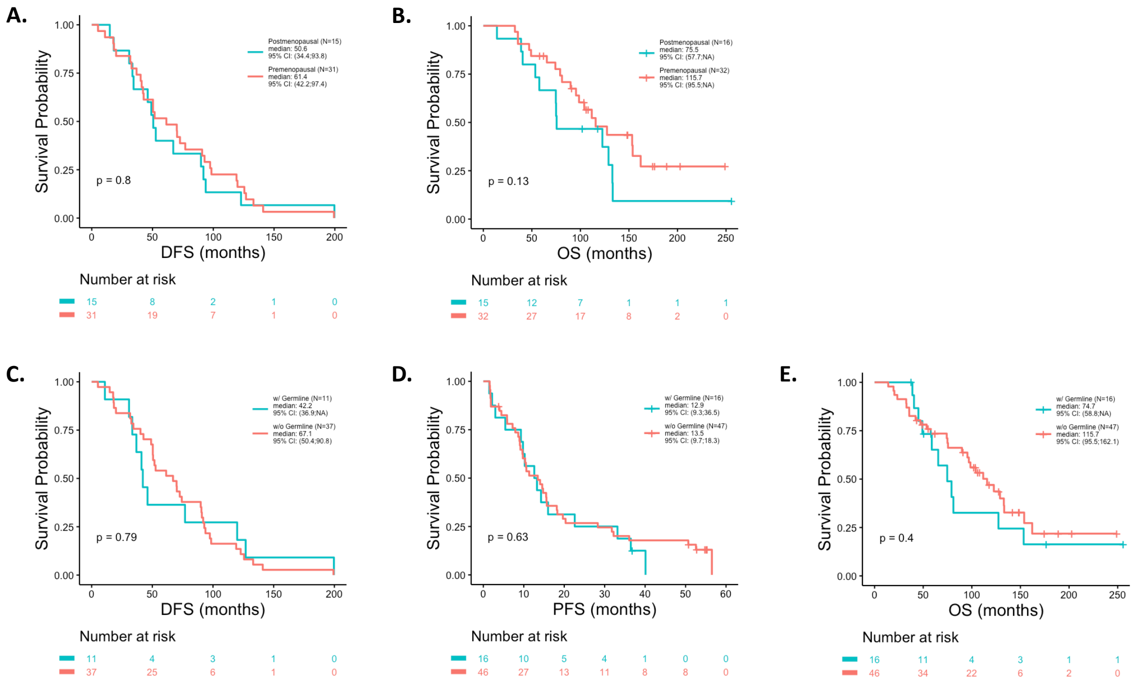

Supplementary Figure 2. Outcomes in association with menopausal and germline mutation status. Kaplan-Meier survival analysis was performed to analyze disease free survival (DFS), progression free survival (PFS) and overall survival (OS) in association with menopausal and germline mutation status. P-values were calculated using the log-rank test, with significance set at $p \leq 0.05$. No significant associations were observed between outcomes (DFS or OS) and menopausal status (**A, B**) or outcomes (DFS, PFS or OS) and germline mutation (**C, D, E**) status.

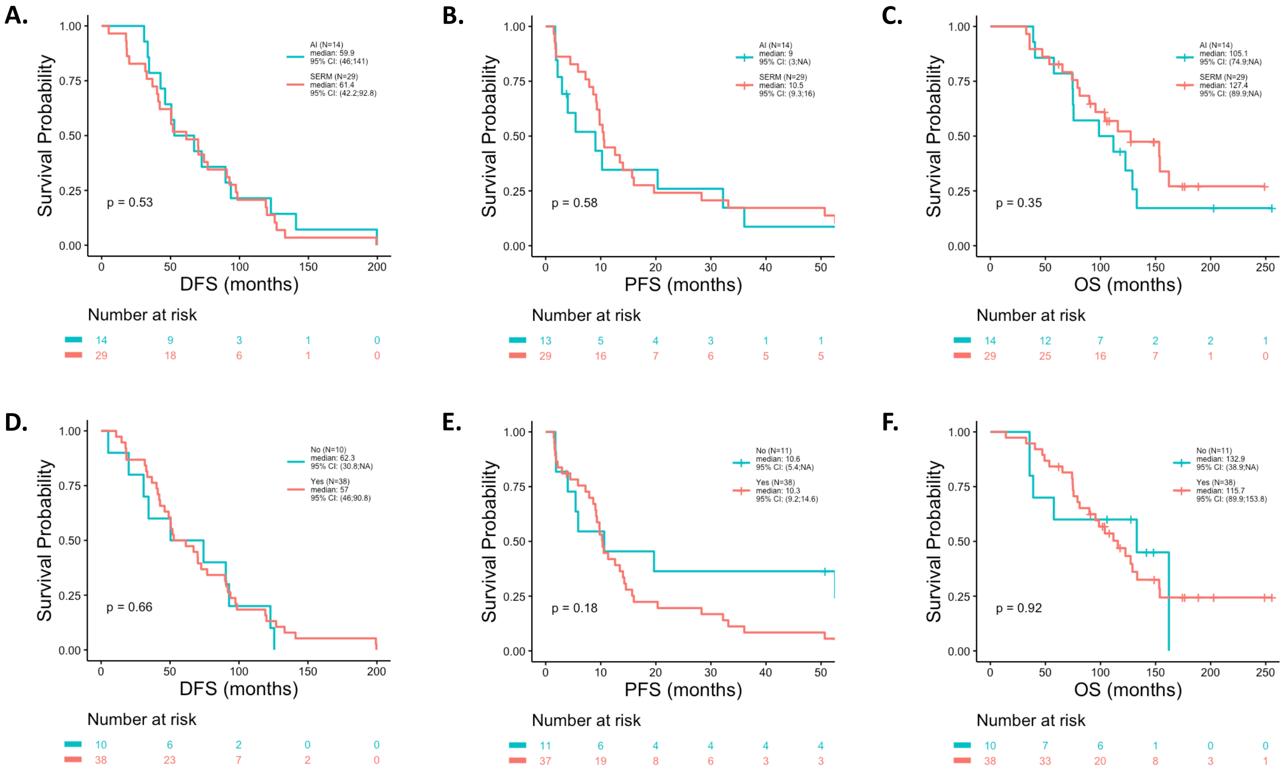

Supplementary Figure 3. Outcomes in association with type of adjuvant endocrine therapy and adjuvant endocrine therapy and receipt of adjuvant chemotherapy. Kaplan-Meier survival analysis was performed to analyze disease free survival (DFS), progression free survival (PFS) and overall survival (OS) in association with type of adjuvant endocrine therapy (AIs vs. SERMS) (**A**,**B**,**C**) or receipt of adjuvant chemotherapy (**D**,**E**,**F**). P-values were calculated using the log-rank test, with significance set at $p \leq 0.05$. No significant associations were observed between outcomes and type of endocrine therapy or between outcomes and receipt of adjuvant chemotherapy.

A.

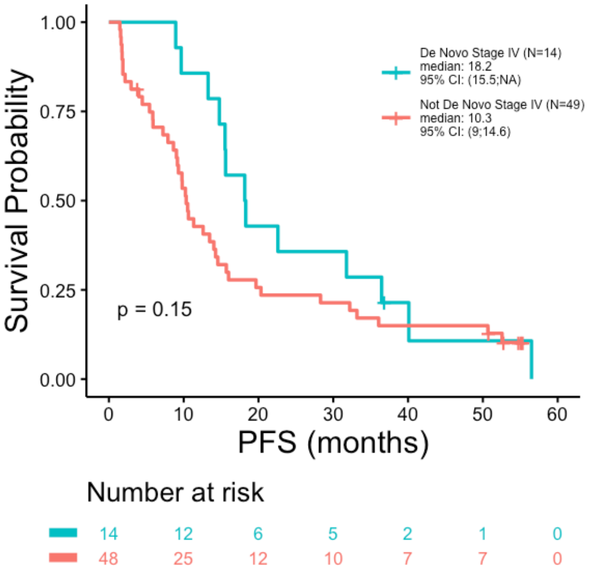

Supplementary Figure 4. Outcomes in association with recurrent vs. Stage IV metastatic disease. Kaplan-Meier survival analysis was performed to analyze progression free survival (PFS) in association with type of Stage IV disease. P-values were calculated using the log-rank test, with significance set at $p \leq 0.05$. While there was a trend for shorter PFS in association with de novo Stage IV disease vs. recurrent Stage IV disease, this trend was not significant.

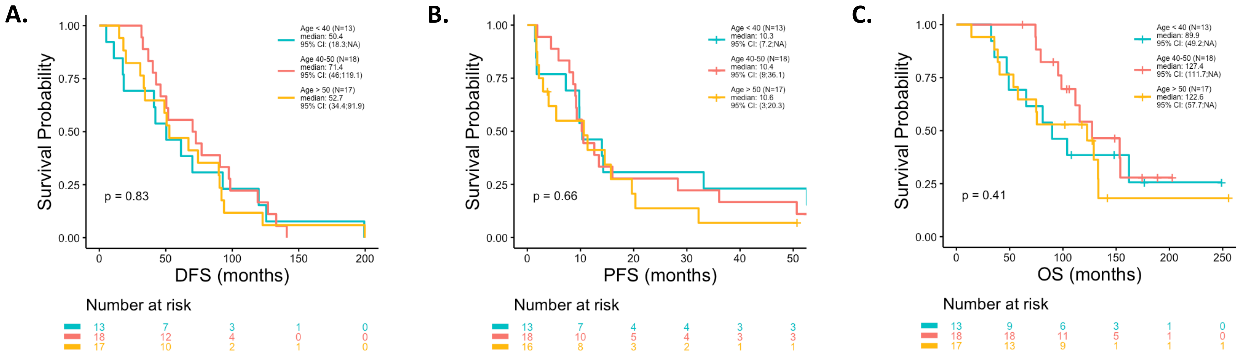

Supplementary Figure 5. Outcomes in association with age in recurrent Stage IV patients. Kaplan-Meier survival analysis was performed to analyzed disease free progression (DFS), progression free survival (PFS) and overall survival (OS) in patients with recurrent Stage IV disease only, excluding

patients with de novo Stage IV disease. P values were calculated using the log-rank test with significance set at $P \leq 0.05$. No significant associations were observed between outcomes and age.
